# Evaluation of Hemoglobin Cutoff Levels to Define Anemia Among Healthy Individuals

**DOI:** 10.1001/jamanetworkopen.2021.19123

**Published:** 2021-08-06

**Authors:** O. Yaw Addo, Emma X. Yu, Anne M. Williams, Melissa Fox Young, Andrea J. Sharma, Zuguo Mei, Nicholas J. Kassebaum, Maria Elena D. Jefferds, Parminder S. Suchdev

**Affiliations:** 1 Nutrition Branch, International Micronutrient Malnutrition Prevention and Control Program Unit, Centers for Disease Control and Prevention, Atlanta, Georgia; 2 Emory University Rollins School of Public Health, Atlanta, Georgia; 3 McKing Consulting Corporation, Atlanta, Georgia; 4 USPHS Commissioned Corps, Atlanta, Georgia; 5 Institute for Health Metrics and Evaluation, University of Washington, Seattle

## Abstract

**Importance:**

Anemia, defined as low hemoglobin (Hb) concentration insufficient to meet an individual’s physiological needs, is the most common blood condition worldwide.

**Objective:**

To evaluate the current World Health Organization (WHO) Hb cutoffs for defining anemia among persons who are apparently healthy and to assess threshold validity with a biomarker of tissue iron deficiency and physiological indicator of erythropoiesis (soluble transferrin receptor [sTfR]) using multinational data.

**Design, Setting, and Participants:**

In this cross-sectional study, data were collected and evaluated from 30 household, population-based nutrition surveys of preschool children aged 6 to 59 months and nonpregnant women aged 15 to 49 years during 2005 to 2016 across 25 countries. Data analysis was performed from March 2020 to April 2021.

**Exposure:**

Anemia defined according to WHO Hb cutoffs.

**Main Outcomes and Measures:**

To define the healthy population, persons with iron deficiency (ferritin <12 ng/mL for children or <15 ng/mL for women), vitamin A deficiency (retinol-binding protein or retinol <20.1 μg/dL), inflammation (C-reactive protein >0.5 mg/dL or α-1-acid glycoprotein >1 g/L), or known malaria were excluded. Survey-specific, pooled Hb fifth percentile cutoffs were estimated. Among individuals with Hb and sTfR data, Hb-for-sTfR curve analysis was conducted to identify Hb inflection points that reflect tissue iron deficiency and increased erythropoiesis induced by anemia.

**Results:**

A total of 79 950 individuals were included in the original surveys. The final healthy sample was 13 445 children (39.9% of the original sample of 33 699 children; 6750 boys [50.2%]; mean [SD] age 32.9 [16.0] months) and 25 880 women (56.0% of the original sample of 46 251 women; mean [SD] age, 31.0 [9.5] years). Survey-specific Hb fifth percentile among children ranged from 7.90 g/dL (95% CI, 7.54-8.26 g/dL in Pakistan) to 11.23 g/dL (95% CI, 11.14-11.33 g/dL in the US), and among women from 8.83 g/dL (95% CI, 7.77-9.88 g/dL in Gujarat, India) to 12.09 g/dL (95% CI, 12.00-12.17 g/dL in the US). Intersurvey variance around the Hb fifth percentile was low (3.5% for women and 3.6% for children). Pooled fifth percentile estimates were 9.65 g/dL (95% CI, 9.26-10.04 g/dL) for children and 10.81 g/dL (95% CI, 10.35-11.27 g/dL) for women. The Hb-for-sTfR curve demonstrated curvilinear associations with sTfR inflection points occurring at Hb of 9.61 g/dL (95% CI, 9.55-9.67 g/dL) among children and 11.01 g/dL (95% CI, 10.95-11.09 g/dL) among women.

**Conclusions and Relevance:**

Current WHO cutoffs to define anemia are higher than the pooled fifth percentile of Hb among persons who are outwardly healthy and from nearly all survey-specific estimates. The lower proposed Hb cutoffs are statistically significant but also reflect compensatory increased erythropoiesis. More studies based on clinical outcomes could further confirm the validity of these Hb cutoffs for anemia.

## Key Points

**Question:**

Are the current World Health Organization (WHO) hemoglobin (Hb) cutoffs to define anemia comparable to statistical and physiological Hb cutoffs calculated using representative surveys from multiple countries collected in the last 20 years?

**Findings:**

In this cross-sectional study of 79 950 Hb observations from population-based surveys that covered all WHO geographical regions, the WHO cutoffs for defining anemia were higher than the fifth percentile of nearly all countries except the US. This finding held for children aged 6 to 59 months and nonpregnant women aged 15 to 49 years and was confirmed using a physiological measure of increased red blood cell production.

**Meaning:**

These findings suggest that lower Hb cutoffs based on pooled multinational data can be considered for defining anemia among children and nonpregnant women.

## Teaser

This cross-sectional study uses multinational data to evaluate the current World Health Organization hemoglobin cutoffs for defining anemia among persons who are apparently healthy.

## Introduction

Anemia, or low hemoglobin (Hb) concentration insufficient to meet an individual’s physiological needs, is the most common blood condition and affects approximately one-third of the world’s population.^1,2^ The World Health Organization (WHO) Hb cutoffs to define anemia^1^ were first established in 1968 by experts who stated that, “over 95% of normal individuals are believed to show Hb levels higher than the cutoffs given” (ie, 11.0 g/dL for children and 12.0 g/dL for nonpregnant women [to convert to grams per liter, multiply by 10]).^3^ Although these cutoffs were derived from a few studies involving European^4,5^ and North American^6,7^ (US and Canada) men, children, and pregnant women, they have been applied uniformly among all geographical regions,^3^ with adjustments for those residing at higher altitudes or who smoke cigarettes.^8^

Evaluation of the WHO Hb cutoffs has been a subject of active research for decades. These cutoffs were derived from statistical cutoffs not linked with physiological or health outcomes. Furthermore, the appropriateness of these cutoffs for defining anemia among certain population groups, age groups, and ethnicities has been questioned repeatedly.^9,10,11,12,13,14,15,16^ There is an urgent need to reexamine global thresholds to define anemia using data from diverse populations and low-income and middle-income countries to inform public health programs. Thus, our objectives were to test the appropriateness of pooling the fifth percentile Hb cutoff levels from different multinational surveys, compare survey-specific and pooled fifth percentile Hb estimates with the current WHO Hb anemia cutoffs, and examine the validity of Hb cutoffs by using a physiological indicator of erythropoiesis or red blood cell production using soluble transferrin receptor (sTfR) ^17,18,19^ among children aged 6 to 59 months and nonpregnant women aged 15 to 49 years.^17,18,19^ This work could have clinical and programmatic implications for anemia screening and control globally.

## Methods

### Analytical Data Sources

We analyzed secondary cross-sectional data collated as part of the Biomarkers Reflecting Inflammation and Nutritional Determinants of Anemia (BRINDA) project. BRINDA included data sets from nationally or regionally representative household nutrition surveys conducted after 2005 with similar sampling and data collection methods, detailed elsewhere.^20^ In addition, we included public domain data from the China Health and Nutrition Survey to further expand the geographical representativeness of our analytical database.^21^ The institutional review boards of the National Institutes of Health and Centers for Disease Control and Prevention reviewed and classified the BRINDA protocol as non–human participants research; thus, informed consent was not needed, in accordance with 45 CFR §46. The Strengthening the Reporting of Observational Studies in Epidemiology (STROBE) reporting guideline was followed.

To be included in the BRINDA database, each survey must have nonmissing Hb, inflammation biomarkers, and a nutritional biomarker of iron (ferritin or transferrin receptor) or vitamin A (retinol or retinol-binding protein [RBP]) data. We evaluated data for children from 24 countries (27 surveys) and women from 23 countries (22 surveys), which included 79 950 individuals (33 699 children and 46 251 women), before applying an inclusion criterion.

### Study Measures, Case Definitions, and Inclusion Criteria

The current WHO anemia cutoffs were established by 1968 WHO guidance and were based on the fifth percentile Hb in smaller studies of Europeans^4,5^ and Canadians^6^ and later validated using a US population.^22,23^ As such, this study also considers the fifth percentile Hb threshold in a multinational sample with individual-level indicators. To assess the survey-specific and pooled fifth percentile Hb threshold, we restricted the analysis to apparently healthy individuals, defined as those who were iron replete (ferritin ≥12 ng/mL for children and ≥15 ng/mL for women [to convert to micrograms per liter, multiply by 1.0])^24^ with no evidence of vitamin A deficiency (RBP or retinol ≥20.1 μg/dL [to convert to micromoles per liter, multiply by 0.0349]),^25^ no inflammation (C-reactive protein ≤0.5 mg/dL [to convert to milligrams per liter, multiply by 10] or α-1-acid glycoprotein ≤1 g/L), or malaria, where measured (eTable 1 and eTable 2 in the Supplement). All surveys were required to have Hb, ferritin, and inflammation, to assess the appropriateness of pooling Hb fifth percentile, but other indicators (vitamin A and malaria) were included only when available.

Ferritin and RBP or retinol concentrations were not inflammation-adjusted because individuals with inflammation were excluded to define the healthy subpopulation. Six surveys with fewer than 100 healthy individuals were excluded from Hb fifth percentile analyses to enable robust percentile estimates. After the exclusions for unhealthy individuals and surveys with fewer than 100 observations, 22 and 21 surveys were used to assess children and women, respectively, for appropriateness of pooling Hb fifth percentile. To examine the validity of the Hb threshold by using a physiological indicator of erythropoiesis, all participants with sTfR and Hb data were included in analyses; there were no restrictions for micronutrient deficiencies, inflammation, or malaria to use the full range of sTfR concentrations (17 surveys for children and 17 for women).

### Statistical Analysis

Statistical analyses were performed with R statistical software version 4.0.1 (R Project for Statistical Computing). Data management was done in SAS statistical software version 9.4 (SAS Institute). Descriptive statistics were unweighted (ie, assumed simple random sampling) because we did not expect the healthy subsample in each survey to be representative of the original design and sampled population. Statistical significance was *P* < .05 with 95% CIs. Linear mixed quantile regression with likelihood-ratio tests and random-effect meta-analysis with restricted maximum likelihood were used to calculate significance. All tests were 2-sided. Hb concentrations were adjusted for altitude following the WHO approach^3^ for all surveys, except China; Pakistan; Bangladesh; Gujarat, India; Cambodia; Côte d’Ivoire; Cameroon; Nigeria; Kenya; Liberia; Philippines; Nicaragua; and the US, which either had no altitude data, or the highest elevation in the country was less than 1000 m above sea level, precluding the need for adjustment.^3^ Hb values were adjusted for smoking among women in surveys with available data on smoking (Colombia, Ecuador, Mexico 2006 and 2012, Great Britain, and the US).

To assess the viability of pooling across surveys to derive fifth percentile Hb, we calculated intraclass correlations (ICCs) around the fifth percentile of Hb with linear quantile mixed models^26^ nesting survey as a random intercept, controlling for age (continuous, children [months] or women [years]) and child sex. To compare survey-specific Hb values to the current WHO Hb threshold for anemia, univariate quantile ranking was used to estimate survey-specific fifth percentile Hb by using the R Software Survey package.^27^ All surveys’ meta-analyzed and pooled estimates were derived using the R metafor^28^ package for each population groups. Heterogeneity across surveys for the fifth percentile Hb was examined by using τ, an estimate of SD,^29^ as derived from meta-analyses of estimates from the individual surveys. Forest plots were used to visualize the survey-specific and the pooled fifth percentile Hb estimates.

For the Hb-for-sTfR erythropoiesis curve analyses, restricted cubic splines^30^ with 5 knots^31^ were used to fit a nonlinear model between Hb and sTfR in both population groups. Ordinary differential equations were then applied to solve for the second-order derivatives (*ΔHb*^2^*/ΔsTfR*^2^*)* at the first 2 inflection points. The 95% CIs around Hb inflection points were obtained from 5000 bootstrap resampling and were optimism bias–corrected by using bias-corrected acceleration.^32^ On the basis of the different stages of iron deficiency (ID), the second inflection is hypothesized to reflect a gradual onset of iron-deficient erythropoiesis, which is characterized by increased bone marrow erythropoietic activity as a compensatory response to decreasing Hb or anemia development from ID.^17,33^ Numerous sensitivity analyses, including using ferritin cutoffs higher than those proposed by the WHO,^24^ were performed, all of which confirmed the robustness of our results (eAppendix, eFigure 1, eFigure 2, and eFigure 3 in the Supplement). Data analysis was performed from March 2020 to April 2021.

## Results

The criteria to identify an apparently healthy population resulted in the exclusion of 44.1% and 60.1% of the available data among children and women, respectively. The range of data loss varied by survey from 17.0% to 100.0% of individuals (eg, 98.0% of respondents from the Burkina Faso data set were excluded, and the survey was dropped because there were <100 observations) (eTable 2 and eTable 3 in the Supplement). Across surveys, the 13 445 children considered to be healthy (39.9% of the original sample; 6750 boys [50.2%]) were, on average, 5.5 months older compared with the overall sample of 33 699 children (mean [SD] age, 32.9 [16.0] months vs 29.9 [15.6] months) (Table 1). The 25 880 women considered to be healthy (56.0% of the original sample) were, on average, 0.2 years younger compared with the overall sample of 46 251 women (mean [SD] age, 31.0 [9.5] years vs 30.9 [9.6] years). Density plots demonstrated that the entire Hb distribution of the healthy subpopulation was right-shifted compared with the overall population, irrespective of adjusting Hb for smoking or altitude, suggesting that apparently healthy and iron-replete individuals were identified (eFigure 1 in the Supplement). The healthy subgroup had lower prevalence of anemia on the basis of WHO cutoffs^1^: 23.4% compared with 40.9% (overall) for children, and 13.0% compared with 22.3% (overall) for women.

**Table 1.** Descriptive Characteristics and Prevalence of Selected Biological Indicators Among the Total Sample and Apparently Healthy Subsample in a Multinational Sample

Characteristic	Participants, No. (%)			
	Preschool children aged 6-59 mo		Nonpregnant women aged 15-49 y	
	Overall (n = 33 699)	Healthy subgroup (n = 13 445)	Overall (n = 46 251)	Healthy subgroup (n = 25 880)
Age, mean (SD), mo for children or y for women	29.9 (15.6)	32.9 (16.0)	31.0 (9.5)	30.9 (9.9)
Sex				
Male	17 391 (51.6)	6750 (50.2)	0	0
Female	16 308 (48.4)	6695 (49.8)	46 251 (100.0)	25 880 (100.0)
Biomarkers and infection, % (95% CI)^a^				
Iron deficiency	22.1 (21.6-22.5)	NA	21.2 (20.8-21.6)	NA
Vitamin A deficiency	29.0 (28.5-29.5)	NA	9.1 (8.7-9.4)	NA
Inflammation	32.7 (32.2-33.3)	NA	21.9 (21.5-22.3)	NA
Malaria	26.0 (24.9-27.0)	NA	12.7 (11.8-13.7)	NA
Anemia	40.9 (40.4-41.4)	23.4 (22.6-24.1)	22.3 (21.9-22.7)	13.0 (12.6-13.4)
Blood draw method				
Venous	14 628 (46.4)	5104 (38.0)	23 759 (52.4)	13 904 (53.7)
Capillary	16 885 (53.6)	8341 (62.0)	21 586 (47.6)	11 976 (46.3)
Hb assessment method				
Automated hematology analyzer	3150 (10.0)	2276 (16.9)	11 733 (25.9)	7883 (30.5)
Hemocue model				
Hb-B	3148 (10.0)	939 (7.0)	863 (1.9)	568 (2.2)
201+	22 925 (72.7)	9277 (69.0)	29 193 (64.4)	14 946 (57.8)
301	2290 (7.3)	956 (7.1)	3556 (7.8)	2486 (9.6)

Abbreviations: Hb, hemoglobin; NA, not applicable.

^a^ Iron deficiency was defined as ferritin less than 12 ng/mL for children or less than 15 ng/mL for women (to convert to micrograms per liter, multiply by 1.0). Vitamin A deficiency was defined as retinol-binding protein or retinol less than 20.1 μg/dL, when available (to convert to micromoles per liter, multiply by 0.0349). Inflammation was defined as C-reactive protein greater than 0.5 mg/dL (to convert to milligrams per liter, multiply by 10) or α-1-acid glycoprotein greater than 1 g/L. Anemia was defined as Hb less than 11.0 g/dL for children, and less than 12.0 g/dL for nonpregnant women (to convert to grams per liter, multiply by 10). Hb values were adjusted for altitude, when available (Afghanistan, Azerbaijan, Colombia, Ecuador, Great Britain, Laos, Malawi, Mexico 2006 and 2012, and Rwanda); otherwise, no adjustment was applied, or if altitude was less than 1000 m, no adjustment was needed. Hb values further adjusted for smoking among women (Colombia, Ecuador, Mexico 2006 and 2012, Great Britain, and US). Healthy defined as no inflammation, no iron deficiency, no vitamin A deficiency, and no known malaria. All estimates were unweighted and were derived from pooled analyses of all surveys. Percentages for vitamin A deficiency and malaria are based only on surveys that measured vitamin A or malaria. Country-specific estimates are provided in eTable 1 and eTable 2 in the Supplement.

The intersurvey ICC around the Hb fifth percentile was low, accounting for 3.6% and 3.5% of the variance among children and women, respectively, which supported the appropriateness of pooling multinational Hb data. Most of the ICC around the Hb fifth percentile was from interindividual variance across surveys (96.4% ICC for children and 96.5% ICC for women) (Table 2). Mean Hb intersurvey ICC explained less than 30% of Hb variance compared with the interindividual variance contribution. The upper tail of the Hb distribution (95th percentile) was consistent with the lower tail, where intersurvey ICC explained approximately 4% of the variance in children and women (data not shown). Table 3 shows results of sensitivity analyses using higher ferritin cutoffs for ID and higher retinol or RBP cutoffs for vitamin A deficiency. In both target groups, no significant differences in the pooled Hb fifth percentile estimate were observed, and there was substantial data loss when using higher ferritin cutoffs.

**Table 2.** Associations Between Age and Sex With Fifth Percentile Hb in a Multinational Sample of Apparently Healthy Individuals^a^

Variable	Factors associated with fifth percentile Hb^b^			
	Preschool children 6-59 mo (n = 13 445)		Nonpregnant women 15-49 y (n = 25 880)	
	β (SE)	*P* value	β (SE)	*P* value
Fixed effects				
Intercept, Hb, g/dL	10.26 (0.32)	<.001	10.76 (0.05)	<.001
Age, y^c^	0.19 (0.02)	<.001	–0.03 (0.4)	.41
Age squared, y^d^	–0.00 (0.0)^e^	.62	0.00 (0.0)^e^	.10
Sex of child	0.28 (0.76)	.66	NA	NA
Variance decomposition (for random effects), %				
ICC between surveys	3.6	<.001	3.5	<.001
ICC between participants across all surveys	96.4		96.5	

Abbreviations: Hb, hemoglobin; ICC, intraclass correlation; NA, not applicable.

SI conversion factor: To convert Hb to grams per liter, multiply by 10.

^a^ Healthy is defined as no inflammation (C-reactive protein ≤0.5 mg/dL [to convert to milligrams per liter, multiply by 10] or α-1-acid glycoprotein ≤1 g/L), no iron deficiency (ferritin <12 ng/mL for children and <15 ng/mL for women [to convert to micrograms per liter, multiply by 1.0]), no vitamin A deficiency (retinol-binding protein or retinol <20.1 μg/dL [to convert to micromoles per liter, multiply by 0.0349], when available), and no known malaria.

^b^ Linear quantile mixed model results are shown.

^c^ Age was mean centered across surveys.

^d^ The age squared term was added to assess curvilinear relations with Hb. Hb values were adjusted for altitude, when available (Afghanistan, Azerbaijan, Colombia, Ecuador, Great Britain, Laos, Malawi, Mexico 2006 and 2012, and Rwanda); otherwise, no adjustment was applied or, if altitude was less than 1000 m, no adjustment was needed. Hb values were further adjusted for smoking among women (Colombia, Ecuador, Mexico 2006 and 2012, Great Britain, and US).

^e^ Values were nonzero but round to 0.00 at 2 decimal points. Coefficient is −0.001 for children and 0.0013 for women.

**Table 3.** Sensitivity Analyses Examining Higher Thresholds to Define Iron and Vitamin A Sufficiency on Pooled Hemoglobin Fifth Percentile Estimates for Healthy Individuals^a^

Inclusion criteria	Analytical sample, participants, No. (% of original No.)	Hemoglobin fifth percentile (95% CI), g/dL^b^
Preschool children (original N = 33 699)		
No inflammation, no VAD (threshold 1), no malaria, and ferritin ≥12 ng/mL (22 surveys)	13 445 (39.9)	9.65 (9.26-10.04)
No inflammation, no VAD (threshold 2), no malaria, and ferritin ≥20 ng/mL (18 surveys)	6752 (20.0)	9.75 (9.29-10.21)
No inflammation, no VAD (threshold 2), no malaria, and ferritin ≥30 ng/mL (16 surveys)	4041 (11.9)	9.83 (9.34-10.32)
Nonpregnant women of reproductive age (original N = 46 251)		
No inflammation, no VAD (threshold 1), no malaria, and ferritin ≥15 ng/mL (22 surveys)	25 880 (56.0)	10.81 (10.35-11.27)
No inflammation, no VAD (threshold 2), no malaria, and ferritin ≥30 ng/mL (22 surveys)	16 285 (35.2)	11.02 (10.59-11.45)
No inflammation, no VAD (threshold 2), no malaria, and ferritin ≥50 ng/mL (22 surveys)	9030 (19.5)	11.03 (10.58-11.49)
No inflammation, no VAD (threshold 2), no malaria, and ferritin ≥100 ng/mL (12 surveys)	2957 (6.3)	11.02 (10.34-11.70)

Abbreviation: VAD, vitamin A deficiency.

SI conversion factor: To convert ferritin to micrograms per liter, multiply by 1.0; hemoglobin to grams per liter, multiply by 10.

^a^ VAD is defined as retinol-binding protein or retinol greater than or equal to 20.1 μg/dL (threshold 1) or greater than or equal to 3.01 μg/dL (threshold 2) (to convert to micromoles per liter, multiply by 0.0349).

^b^ Estimates are not statistically significant at α = .05 within each target population.

**Figure 1.**
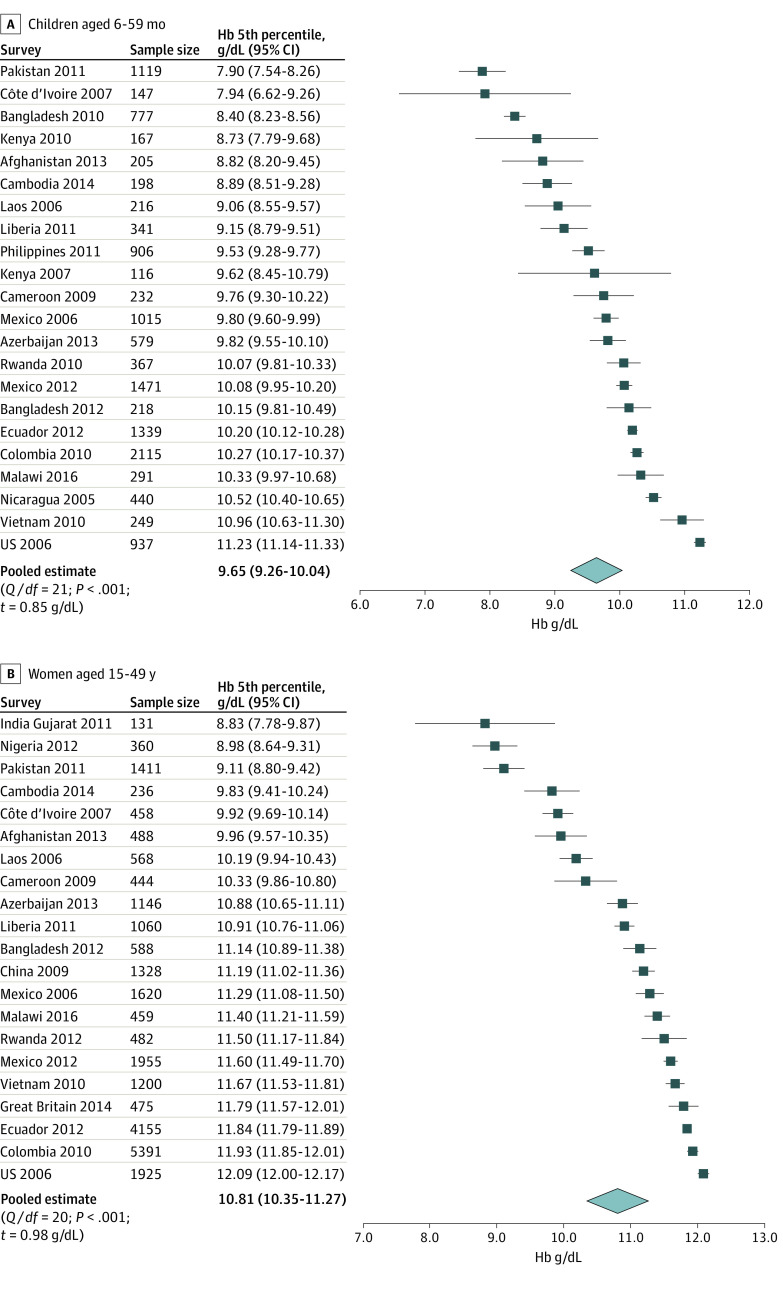
Forest Plots Showing Survey-Specific and Pooled Fifth Percentile (95% CI) of Hemoglobin (Hb) in a Multinational Sample of 13 445 Apparently Healthy Preschool Children Aged 6 to 59 Months and 25 880 Nonpregnant Women Aged 15 to 49 Years Healthy is defined as no inflammation (C-reactive protein ≤0.5 mg/dL [to convert to milligrams per liter, multiply by 10] or α-1-acid glycoprotein ≤1 g/L), no iron deficiency (ferritin <12 ng/mL for children and <15 ng/mL for women [to convert to micrograms per liter, multiply by 1.0]), no vitamin A deficiency (retinol-binding protein or retinol <20.1 μg/dL [to convert to micromoles per liter, multiply by 0.0349], when available), and no known malaria. SEs (95% CIs) around Hb fifth percentile were based on the Wald SE of the estimated proportion below the quantile at a design effect of 1 for simple random sampling. Hb values were adjusted for altitude, when available (Afghanistan, Azerbaijan, Colombia, Ecuador, Great Britain, Laos, Malawi, Mexico 2006 and 2012, and Rwanda); otherwise, no adjustment was applied or the altitude was less than 1000 m, so no adjustment was needed. Hb values were further adjusted for smoking among women (Colombia, Ecuador, Mexico 2006 and 2012, Great Britain, and US). World Health Organization Hb cut points for anemia are 11.00 g/dL for children and 12.00 g/dL for nonpregnant women. To convert Hb to grams per liter, multiply by 10. *Q* / *df* indicates test of Cochrane *Q* statistic for heterogeneity at the given *df*.

Figure 1 displays forest plots of fifth percentile Hb concentrations by survey for apparently healthy children and women. The pooled meta-analyzed fifth percentile Hb estimate for healthy children was 9.65 g/dL (95% CI, 9.26-10.04 g/dL), 1.35 g/dL lower than the WHO cutoff of 11.0 g/dL. Among children, the survey-specific fifth percentile Hb estimates ranged from 7.90 g/dL (95% CI, 7.54-8.26 g/dL) in Pakistan to 11.23 g/dL (95% CI, 11.14-11.33 g/dL) in the US. The pooled meta-analyzed fifth percentile Hb estimate for healthy women was 10.81 g/dL (95% CI, 10.35-11.27 g/dL), 1.19 g/dL lower than the WHO cutoff of 12.0 g/dL. Among women, the survey-specific fifth percentile Hb estimates ranged from 8.83 g/dL (95% CI, 7.77-9.88 g/dL) in Gujarat, India, to 12.09 g/dL (95% CI, 12.00-12.17 g/dL) in the US. The survey-specific fifth percentile Hb threshold among women in China was 0.90 g/dL lower than women in the US (11.19 vs 12.09 g/dL). One-way quantile analyses^34^ comparing each survey fifth percentile Hb against the current WHO anemia cutoffs (11.0 g/dL for children and 12.0 g/dL for women) indicated that most surveys had statistically lower fifth percentile Hb (eTable 4 in the Supplement).

Participant age was significantly associated with Hb at the fifth percentile (β = 0.20; *P* < .001) in children but not women. Additional sensitivity analyses indicated an increasing age-gradient in Hb among children such that those aged 6 to 11 months had lower Hb levels than those older than 48 months (–0.92 g/dL; 95% CI, –1.02 to –0.83 g/dL; *P* < .001) after adjustment for sex, Hb assessment method, and survey. This same sensitivity model showed that after accounting for survey, age, and child sex, neither blood source (venous or capillary) nor assessment method (automated hematology analyzer or not) was independently associated with Hb (eTable 5 in the Supplement).

**Figure 2.**
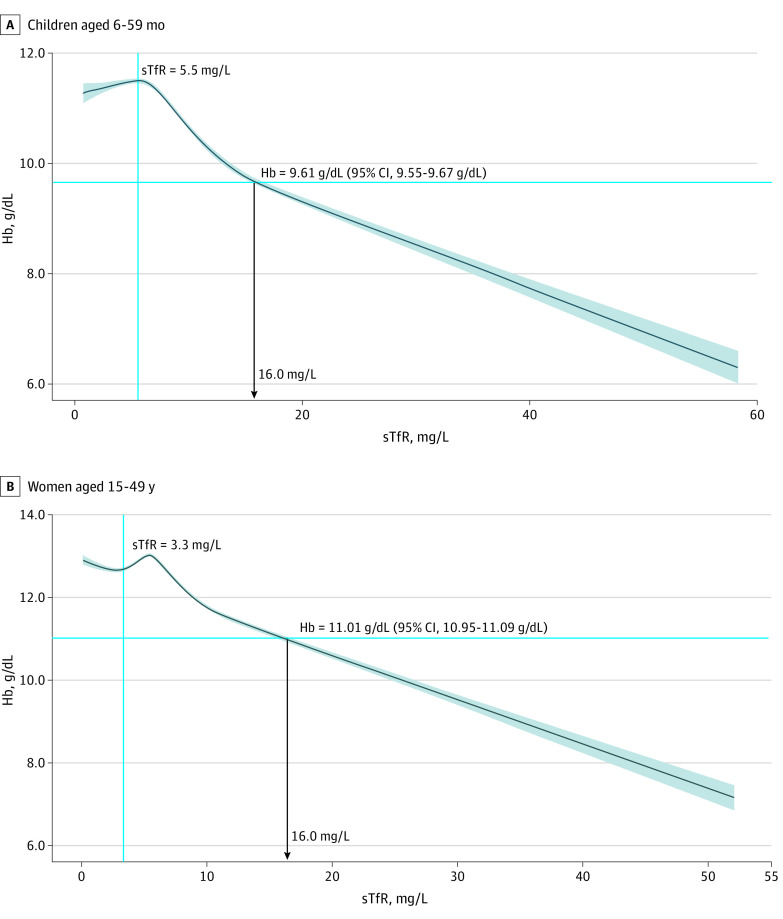
Restricted Cubic Spline Curve Analyses for the Association Between Hemoglobin (Hb) and Soluble Transferrin Receptor (sTfR) Concentrations Among 14 854 Preschool Children Aged 6 to 59 Months and 24 198 Nonpregnant Women Aged 15 to 49 Years Hb values were adjusted for altitude, when available (Afghanistan, Azerbaijan, Colombia, Ecuador, Great Britain, Laos, Malawi, Mexico 2006 and 2012, and Rwanda); otherwise, no adjustment was applied, or the altitude was less than 1000 m, so no adjustment was needed. Hb values were further adjusted for smoking among women (Colombia, Ecuador, Mexico 2006 and 2012, Great Britain, and US). The 95% CIs around Hb inflection points were obtained from 5000 bootstrap resampling and were bias corrected using bias-corrected acceleration. The shaded region around the curve indicates the 95% confidence bands. Analyses were based on all participants with nonmissing sTfR concentrations and Hb. To convert Hb to grams per liter, multiply by 10.

Figure 2 shows Hb-for-sTfR restricted cubic splines curve analysis for participants with Hb and sTfR data. This Hb-sTfR curve revealed distinct phases and clear negative curvilinear associations with inflection points (nonlinear *P* for trend <.001). The initial inflection in sTfR, which reflects tissue ID,^17,33^ occurred at an inflection point of 5.5 mg/L for children and 3.3 mg/L for women (the sTfR values are based on the Ramco assay, as discussed in the eAppendix in the Supplement). The second inflection point of the fitted equation for children (ie, second derivative of Hb with respect to sTfR) occurred at Hb of 9.61 g/dL (95% CI, 9.55-9.67 g/dL) and for women at Hb of 11.01 g/dL (95% CI, 10.95-11.09 g/dL). Results of the Hb-sTfR curve analyses closely matched the meta-analyzed pooled Hb fifth percentile estimate.

## Discussion

By use of data from apparently healthy individuals from 27 surveys representing all WHO geographical regions, we found that the calculated multinational estimates of Hb fifth percentile were 1.35 and 1.19 g/dL lower than the current WHO Hb cutoffs for defining anemia among preschool children and nonpregnant women, respectively. Aside from 2 countries, the survey-specific fifth percentile Hb estimates were significantly lower than the WHO cutoffs for anemia. Intersurvey variance around the Hb fifth percentile was less than 4%, which supports the appropriateness of pooling individual Hb data from a multinational sample and deriving a single-population, group-specific cutoff. The statistical fifth percentile Hb pooled results were reinforced by the physiological association between Hb and increased sTfR concentrations (reflective of iron-deficient erythropoiesis) at Hb concentrations very similar to what was derived from the pooled fifth percentile Hb analysis for each population group.^35^ Our findings were not affected when using higher ferritin cutoffs^36^ for defining ID, which highlights the robustness of these multinational Hb cutoffs for individuals with marked variations in normative Hb and ferritin concentrations.

The current WHO cutoff levels were derived from mainly White adults^37^ but were validated in a multiethnic sample from a single country (US).^22,23^ The results of our multinational pooled fifth percentile Hb estimates were consistent with several studies^10,12,13,14,15,38^ that have called for a downward revision of the Hb cutoffs by approximately 1.0 g/dL for a variety of reasons, including a recent 2021 publication by Sachdev et al^38^ that suggests the use of lower Hb cutoffs to define anemia in children using data from the 2016 India Comprehensive National Nutrition Survey. However, these prior studies were based on examination of effect sizes from published research or data from individual countries. Our study advances knowledge of Hb distributions across countries as we analyzed expansive individual-level data sets that included Hb, micronutrient biomarkers, infection (malaria), and inflammation.^39^ We were able to leverage these biomarkers to identify an apparently healthy subpopulation, which is unique compared with prior validation research of the WHO Hb cutoffs that were limited to iron status of US participants.^22,23^ The low intersurvey variance around the lower tail of the fifth percentile Hb distribution may be explained by anemia etiology, which is multifactorial but may be similar across countries.^40,41^ We adjusted Hb for altitude and smoking (among women),^8,42^ when data were available, thereby reducing their confounding role on Hb. There was low intersurvey variance when analyzing individual-level Hb data of 39 325 apparently healthy individuals, but high interstudy heterogeneity from meta-analysis highlighting the limitation of meta-analyses to directly address this study objective.

Anemia screening in clinical practice and public health surveillance guides programs and interventions.^43^ Where possible, Hb cutoffs defining anemia and its severity should be guided by functional and clinically relevant outcomes. Although clinical outcomes were not available in this work, we modeled Hb to sTfR concentrations, which physiologically reflect erythropoiesis.^44,45,46^ The curves of Hb-sTfR concentrations indicate distinct curvilinear and linear erythropoietic drive phases when Hb values are below a threshold (9.61 g/dL in children and 11.01 g/dL in women) (Figure 2). The linear erythropoietic drive phase describes the expected compensatory physiological response to anemia, including increased tissue demand for iron and increased erythroid in the bone marrow.^17,46,47^ The physiological Hb-sTfR curves support the use of a pooled multinational Hb fifth percentile for defining anemia, as opposed to adopting Hb estimates that are specific to a survey, country, or race/ethnicity,^9^ which could lead to proliferation of multiple different Hb cutoffs and, thus, complicate their clinical application and global disease burden quantification, among other factors. We found that in both preschool children and women, regardless of Hb distribution, the population fifth percentile Hb was lower than 9.61 g/dL and 11.01 g/dL, indicating that tissue ID has already ensued (Figure 2). The Hb fifth percentile derived in this analysis may reflect the development of anemia apparently caused by ID. A cautious interpretation is needed when using Hb alone to identify anemia and guide candidate interventions, as evaluating the factors associated with the development of anemia beyond ID, such as malaria (in endemic regions), vitamin A, vitamin B_12_, folate, and inherited blood disorders, is essential to guide anemia management.

### Strengths and Limitations

The strengths of our work include the use of a large data set of household-based nutrition surveys with biomarkers of ID, vitamin A deficiency, inflammation, and malaria from healthy and diverse populations from multiple geographical regions. Furthermore, we excluded participants with known select proximal factors associated with risk of anemia to identify apparently healthy persons. Similarly, the physiological Hb-for-sTfR curve analyses yielded Hb thresholds similar to those generated from the fifth percentile of population-specific Hb estimates among children and women. We calculated the Hb fifth percentile from the empirical distribution for robust estimates rather than using close-form expressions (of means and SD),^10,22,23^ both of which can be affected by Hb measurement issues across surveys. The application of piecewise cubic spline equations enabled complex sTfR-Hb relationships to be captured.

This study also has limitations that should be considered. Data were cross-sectional, so we were unable to examine temporality among indicators and other factors. Another limitation was that laboratory assessment of Hb was not uniform. Limitations of Hb assessments and blood sampling have been associated with varied anemia prevalence estimates.^48,49,50,51,52,53,54^ In our data, capillary vs venous blood draw was not associated with assignment of the healthy status in the overall population, and among the healthy subpopulation, blood sampling was not associated with Hb, inflammation, or vitamin A concentrations (data not shown). Most of the surveys used point-of-care HemoCue analyzers, except 4 surveys that used automated hematology analyzers. Compared with the less-portable automated hematology analyzers, HemoCue machines might be subject to more variations in measured Hb because of preanalytical and analytical factors in field settings.^55^ Nevertheless, in 2 surveys that both used venous blood and the automated hematology analyzers, the survey-specific fifth percentile Hb threshold among women in China was still 0.90 g/dL lower than women in the US (11.19 vs 12.09 g/dL). It is unclear whether assessment method, blood source, or other analytical factors could explain away the observed differences between the highest vs lowest fifth percentile Hb cutoffs among surveys. Developing best practices for Hb measurement in clinical laboratories and field studies are important priorities of national and global public health agencies.^37^

We defined a healthy population according to iron and vitamin A status, inflammation, and malaria, but were unable to examine other factors associated with anemia, such as inherited blood disorders (eg, sickle cell Hb or thalassemia diagnosed using red blood cell indices such as mean corpuscular volume, or direct laboratory assessment). In addition, some biomarkers of micronutrient status (eg, vitamin A, vitamin B_12_, and folate) and reticulocyte Hb content transferrin saturation were either not available for all surveys or were not measured in any of the population-based surveys. Additional research is needed to examine Hb thresholds for other target groups (eg, younger children, pregnant women, or the elderly) and to further explore the potential utility of age-specific and sex-specific Hb cutoffs in children. The healthy inclusion criteria led to exclusion of a large proportion of data, highlighting widespread micronutrient deficiencies and inflammation. No clinical data from hospitals or medical records were used in this analysis, so we were unable to examine possible associations between Hb and clinical outcomes. Recognizing that the Hb-sTfR curve may represent a biological relationship, we acknowledge that thresholds for anemia based on detrimental health and functional outcomes (eg, exacerbation of underlying clinical conditions, reduced work capacity, fatigue, sleep disturbance, prematurity or low birth weight, or impaired child cognitive development) would advance the field.^56,57,58,59,60^

## Conclusions

On the basis of data from more than 39 000 individuals from 25 countries examined, the current WHO hemoglobin cutoff levels for defining anemia among preschool children and nonpregnant women were found to be significantly higher than the fifth percentile of Hb in apparently healthy individuals from most countries evaluated. Future studies focusing on Hb thresholds associated with functional and clinical health outcomes will improve the understanding of overall disease burden. Until then, revising Hb cutoff level definitions according to pooled multinational data could be considered.

## Notes

Supplement. **eAppendix.** Supplemental Methods **eFigure 1.** Hemoglobin (Hb) Distributions for Apparently Healthy and Overall Sample by Target Group With and Without Altitude and Smoking Adjustment **eFigure 2.** Examination of Hb Assessment Method and Source of Blood on 5^th^ Percentile (95% CI) of Hb Values in an Apparently Healthy Multinational Sample of Individuals **eFigure 3.** Sensitivity Analyses Examining the Impact of Complex Survey Design Effects and Sampling Weights vs Unweighted Simple Random Sampling on 5th Percentile (95% CI) of Hb Values in an Apparently Healthy Multinational Sample of Individuals **eTable 1.** Blood Sampling, Biomarkers and Laboratory Assessment Methods Across Surveys **eTable 2.** Included Surveys and Participant Descriptive Characteristics: Preschool Children Aged 6-59 Months **eTable 3.** Included Surveys and Participant Descriptive Characteristics: Non-Pregnant Women of Reproductive Aged 15-49 y **eTable 4.** Results of One-Way Quantile Test Comparing Survey-Specific 5th Percentile Hb With Current WHO Hb Cutoffs for Defining Anemia in a Multinational Sample of Apparently Healthy Individuals **eTable 5.** Associations Between Hb Concentrations With Participant Age, Hb Analyzer, Type of Blood Sampling in a Multinational Sample of Apparently Healthy Individuals
