# Supplementary material for: Evaluation of Hemoglobin Cutoff Levels to Define Anemia Among Healthy Individuals
Source: JAMA Netw Open. 2021 Aug 6;4(8):e2119123. doi: 10.1001/jamanetworkopen.2021.19123 (PMC8346941; doi:10.1001/jamanetworkopen.2021.19123)
Supplement: Supplement. — eAppendix. Supplemental Methods eFigure 1. Hemoglobin (Hb) Distributions for Apparently Healthy and Overall Sample by Target Group With and Without Altitude and Smoking Adjustment eFigure 2. Examination of Hb Assessment Method and Source of Blood on 5th Percentile (95% CI) of Hb Values in an Apparently Healthy Multinational Sample of Individuals eFigure 3. Sensitivity Analyses Examining the Impact of Complex Survey Design Effects and Sampling Weights vs Unweighted Simple Random Sampling on 5th Percentile (95% CI) of Hb Values in an Apparently Healthy Multinational Sample of Individuals eTable 1. Blood Sampling, Biomarkers and Laboratory Assessment Methods Across Surveys eTable 2. Included Surveys and Participant Descriptive Characteristics: Preschool Children Aged 6-59 Months eTable 3. Included Surveys and Participant Descriptive Characteristics: Non-Pregnant Women of Reproductive Aged 15-49 y eTable 4. Results of One-Way Quantile Test Comparing Survey-Specific 5th Percentile Hb With Current WHO Hb Cutoffs for Defining Anemia in a Multinational Sample of Apparently Healthy Individuals eTable 5. Associations Between Hb Concentrations With Participant Age, Hb Analyzer, Type of Blood Sampling in a Multinational Sample of Apparently Healthy Individuals [file jamanetwopen-e2119123-s001.pdf]

Supplemental Online Content

Addo OY, Yu EX, Williams AM, et al. Evaluation of hemoglobin cutoff levels to define anemia among healthy individuals. *JAMA Netw Open*. 2021;4(8):e2119123. doi:10.1001/jamanetworkopen.2021.19123

eAppendix. Supplemental Methods

eFigure 1. Hemoglobin (Hb) Distributions for Apparently Healthy and Overall Sample by Target Group With and Without Altitude and Smoking Adjustment

eFigure 2. Examination of Hb Assessment Method and Source of Blood on 5th Percentile (95% CI) of Hb Values in an Apparently Healthy Multinational Sample of Individuals

eFigure 3. Sensitivity Analyses Examining the Impact of Complex Survey Design Effects and Sampling Weights vs Unweighted Simple Random Sampling on 5th Percentile (95% CI) of Hb Values in an Apparently Healthy Multinational Sample of Individuals

eTable 1. Blood Sampling, Biomarkers and Laboratory Assessment Methods Across Surveys

eTable 2. Included Surveys and Participant Descriptive Characteristics: Preschool Children Aged 6-59 Months

eTable 3. Included Surveys and Participant Descriptive Characteristics: Non-Pregnant Women of Reproductive Aged 15-49 y

eTable 4. Results of One-Way Quantile Test Comparing Survey-Specific 5th Percentile Hb With Current WHO Hb Cutoffs for Defining Anemia in a Multinational Sample of Apparently Healthy Individuals

eTable 5. Associations Between Hb Concentrations With Participant Age, Hb Analyzer, Type of Blood Sampling in a Multinational Sample of Apparently Healthy Individuals

This supplemental material has been provided by the authors to give readers additional information about their work.

© 2021 Addo OY et al. *JAMA Network Open.*

eAppendix. Supplemental Methods

Laboratory Methods

Venous or capillary blood was collected in the 30 surveys (not all had both target groups) examined. Hb was assessed with automated hematology analyzers in 4 surveys (US, Great Britain, China, and Ecuador) and point-of-care Hemocue in 26 surveys (i.e., thirteen Hemocue model 201+, four Hemocue model 301, four Hemocue model Hb-B, and 5 surveys did not specify the Hemocue model number). Ferritin, soluble transferrin receptor (sTfR), retinol, retinol binding protein (RBP), and inflammatory biomarkers (alpha-1-acid glycoprotein [AGP] or C-reactive protein [CRP]) were also assessed, although the selection of biomarkers varied by survey (**eTable 1**). Ferritin, sTfR, RBP, CRP, and AGP were measured with ELISA or immunoassay techniques, and retinol was assessed with high-performance liquid chromatography (HPLC).[61, 62] sTfR values were based on Ramco assay or converted from Roche to Ramco assay concentrations. Malaria was assessed with rapid test kits based on malaria antigen (HRP2/pLDH) combo for *Plasmodium falciparum* and *Plasmodium Vivax*.

Sensitivity Analyses

First, for the Hb threshold objective, we first compared Hb distributions by using density plots to assess whether the distribution of the overall population Hb was left-shifted compared to the healthy subsample. Second, to assess the appropriateness of pooling data of healthy individuals, we examined ICC at other quantiles (at mean, and 95th percentile) of Hb distribution by using mixed models. Third, we tested for the equality of pooled effect sizes for survey-specific derived 5th percentile Hb values contrasted against pooled values derived from the Hb assessment method stratified analyses (i.e., automated hematology analyzer, Hemocue model 201+, Hemocue 301, Hemocue-Hb-B, or Hemocue but no model number listed: survey-stratified vs Hb method or blood

© 2021 Addo OY et al. *JAMA Network Open.*

source stratified estimates). Fourth, we evaluated if the Hb assessment method or the blood source (venous or peripheral/capillary) was associated with 5th percentile Hb concentrations by using mixed models with the surveys as random effects. Fifth, because ferritin <12 ng/mL and <15 ng/mL has been shown to have low sensitivity and specificity in comparison to bone marrow assessment (the gold standard), therefore we examined results by using higher cutoffs to define ID (with ferritin <20 ng/mL & 30 ng/mL for children; and 30, ng/mL, 50 ng/mL, and 100 ng/mL for women)[36, 63] and VAD for apparently healthy children and women (RBP or retinol <1.05 µmol/L [to convert to micromoles per deciliter, divide by 0.0349]).[25] Sixth, we conducted Hb 5th percentile and meta-analyses by using the original survey sampling weights with complex design effects, where applicable, and we compared results with unweighted analysis (with SRS).

As shown in table 3 and supplemental figures 1-3, all sensitivity analyses regarding the pooled 5th percentile Hb (objective a and b) suggested that our results are robust. Further, Table 3 indicated neither the use of higher thresholds to define ID and VAD nor Hb assessment method and blood source significantly alter the pooled 5th percentile Hb (**eFigure 2**). Analyses accounting for complex survey design (i.e., cluster, strata, weight) yielded pooled 5th percentile Hb estimates that were not statistically different from the unweighted analyses that assumed SRS (**eFigure 3**).

© 2021 Addo OY et al. *JAMA Network Open.*

eFigure 1. Hemoglobin (Hb) Distributions for Apparently Healthy and Overall Sample by Target Group With and Without Altitude and Smoking Adjustment

Children (Overall n= 33,699; healthy n=13,445)	Non-Pregnant Women (Overall n=46,251; healthy n=25,880)
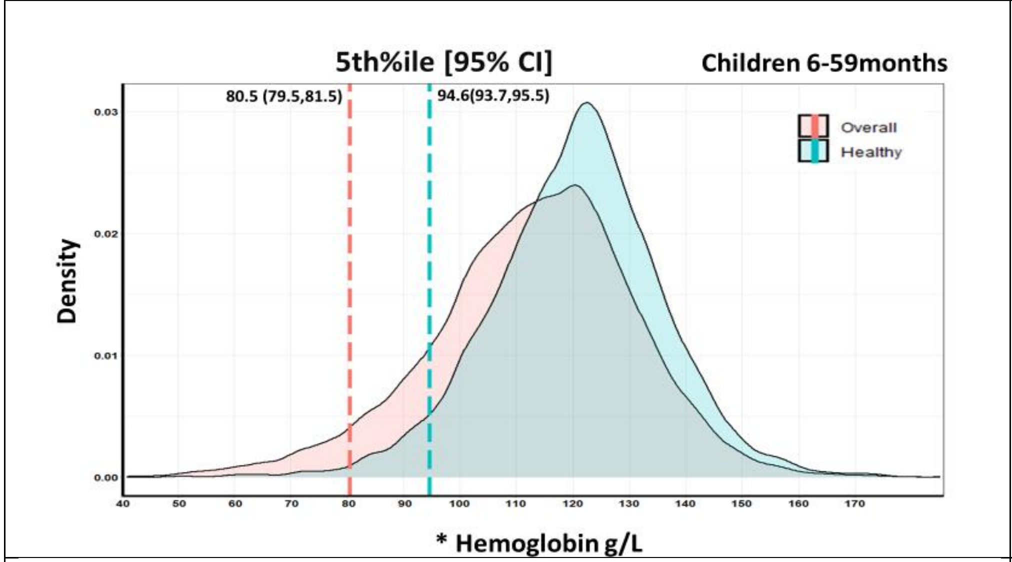	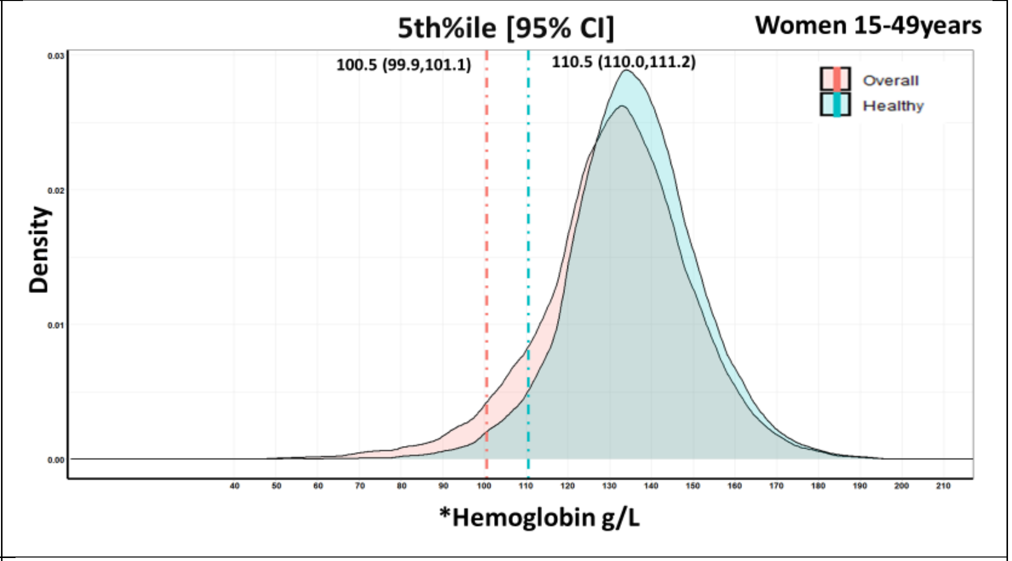
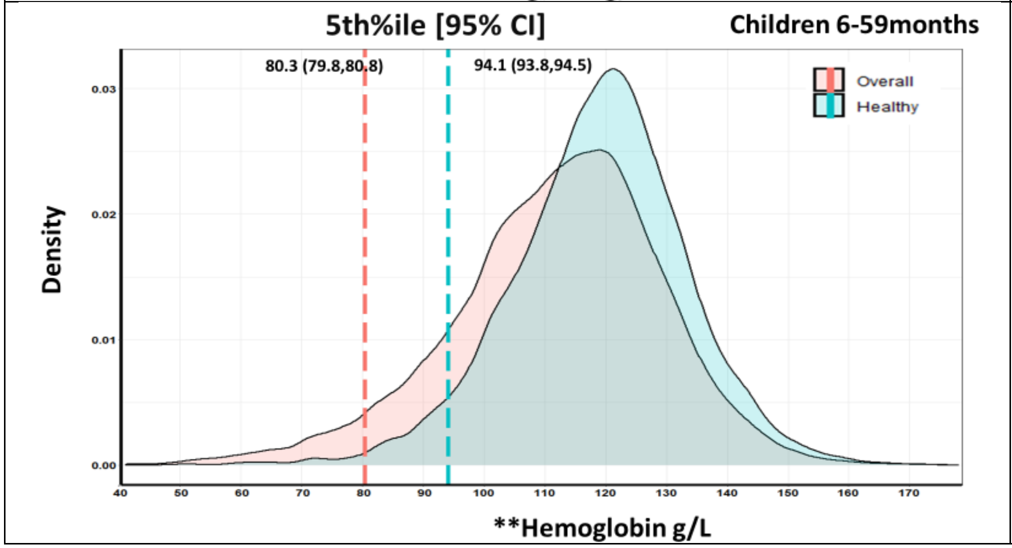	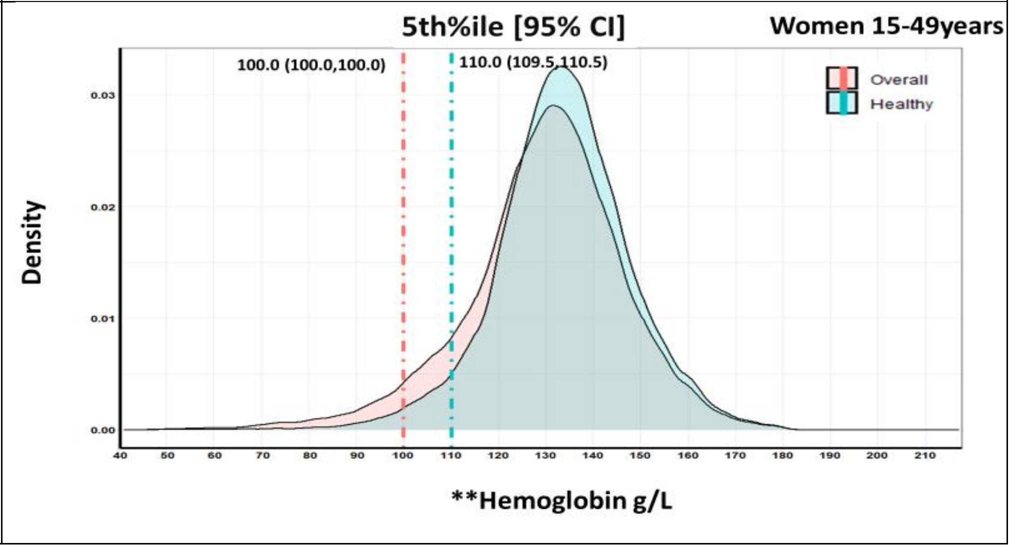

*Unadjusted Hb values (to covert Hb to grams per deciliter, divide by 10). **Hb adjusted for 1) altitude when available (Afghanistan, Azerbaijan, Colombia, Ecuador, Great Britain, Laos, Malawi, Mexico 2006 and 2012, and Rwanda) otherwise no adjustment was applied or <1000m so no adjustment needed, and 2) smoking (Colombia, Ecuador, Mexico 06 and 2012, Great Britain and USA). Healthy defined as no inflammation (CRP ≤ 5 mg/L or AGP ≤ 1 g/L), no iron deficiency (based on ferritin <12 ng/mL for children and <15 ng/mL for women), no vitamin A deficiency (based on RBP or retinol ≥20.1 µg/dL [to convert to micromoles per liter, multiply by 0.0349]) when available, and no known malaria.

© 2021 Addo OY et al. *JAMA Network Open.*

eFigure 2: Examination of Hb Assessment Method and Source of Blood on 5th Percentile (95% CI) of Hb Values in an Apparently Healthy Multinational Sample of Individuals

Apparently Healthy Children (N=13,445)	Apparently Healthy Non-Pregnant Women(N=25,880)

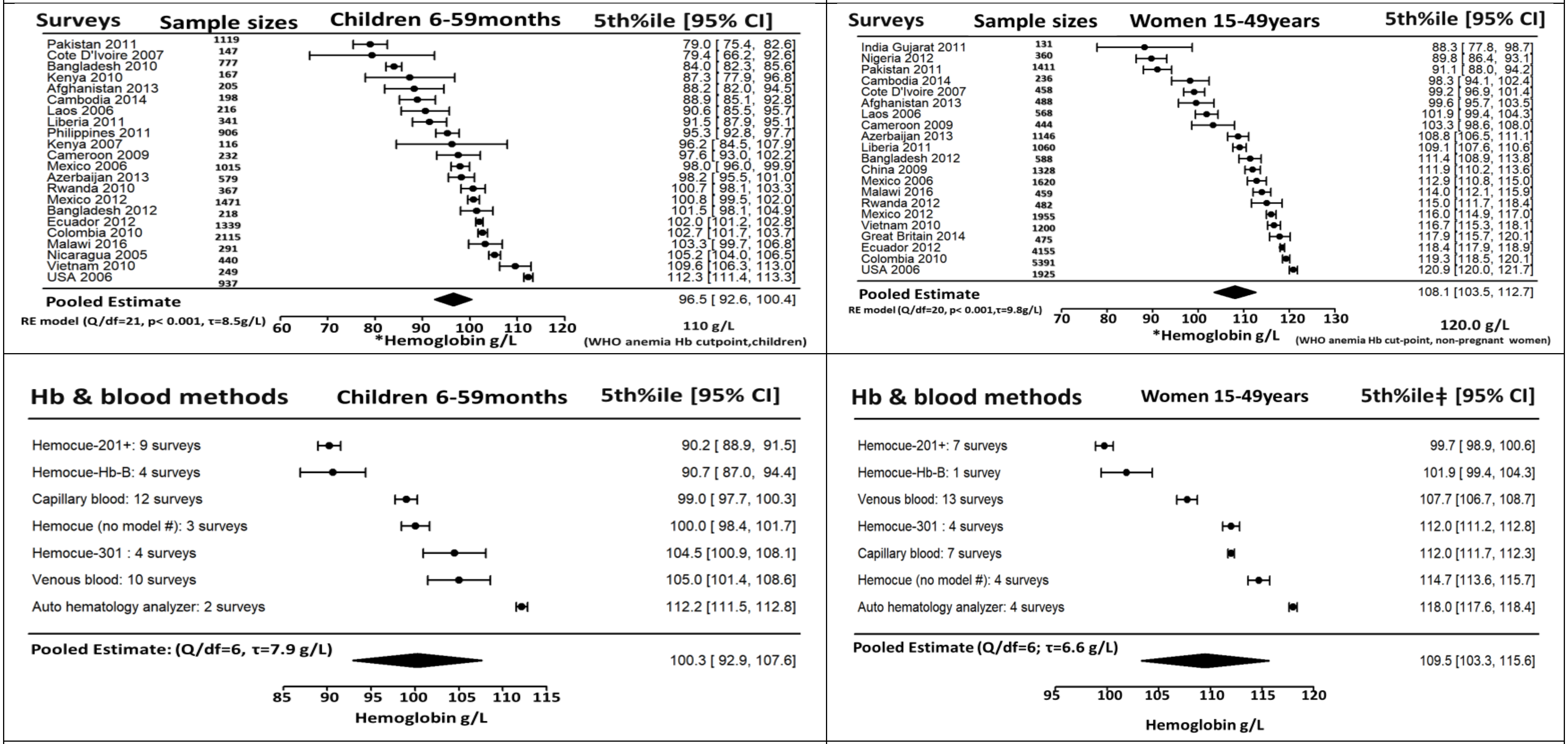

Test of equality of pooled effect sizes: 96.5 =100.3, g/L, p = 0.31	Test of equality of pooled effect sizes:108.1 =109.5 g/L, p= 0.61

Abbreviations and definitions: Healthy defined as no inflammation (C-reactive protein ≤ 5 mg/L or alpha-1acid glycoprotein ≤1 g/L), no iron deficiency (based on ferritin <12 ng/mL for children and <15 ng/mL for women), no vitamin A deficiency (based on RBP or retinol ≥20.1 µg/dL [to convert to micromoles per liter, multiply by 0.0349], when available), and no known malaria. RE, random effects model. Q/df, Test of Cochrane's Q statistic for heterogeneity at the given degrees of freedom (DF). For HemoCue and blood source stratified analyses, count of surveys labeled on the Forest plots are based on healthy subpopulation, after data exclusions (i.e. slightly different from counts on supplemental Table 1).

Standard errors, SE (and 95% CI) around Hb 5%ile were based on the Wald SE of the estimated proportion below the quantile at a design effect of 1 (for SRS). Hb values (to convert Hb to grams per deciliter, divide by 10) were adjusted for attitude when available (Afghanistan, Azerbaijan, Colombia, Ecuador, Great Britain, Laos, Malawi, Mexico 2006 and 2012, and Rwanda) otherwise no adjustment was applied or <1000m so no adjustment needed. Hb values further adjusted for smoking among women (Colombia, Ecuador, Mexico 2006 and 2012, Great Britain and USA).

© 2021 Addo OY et al. *JAMA Network Open.*

eFigure 3: Sensitivity Analyses Examining the Impact of Complex Survey Design Effects and Sampling Weights vs Unweighted Simple Random Sampling on 5th Percentile (95% CI) of Hb Values in an Apparently Healthy Multinational Sample of Individuals.

Apparently Healthy Children (N=13,445)	Apparently Healthy Non-Pregnant Women(N=25,880) -
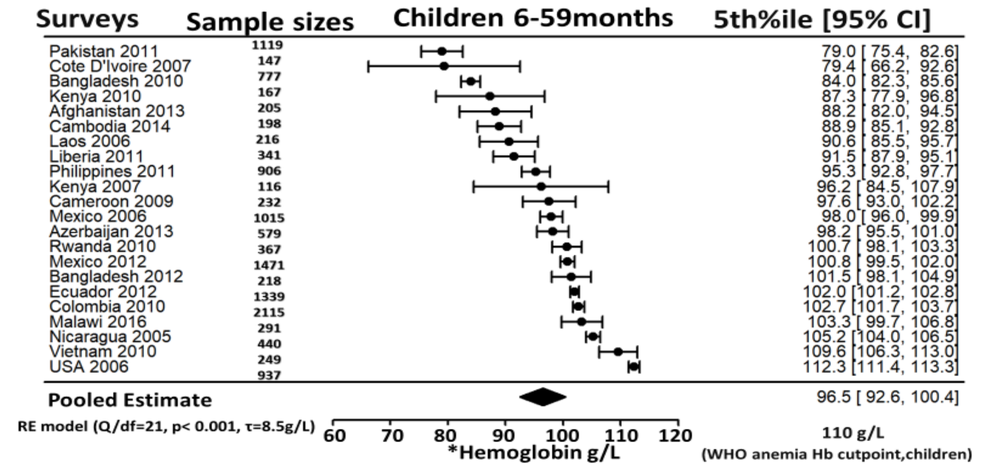	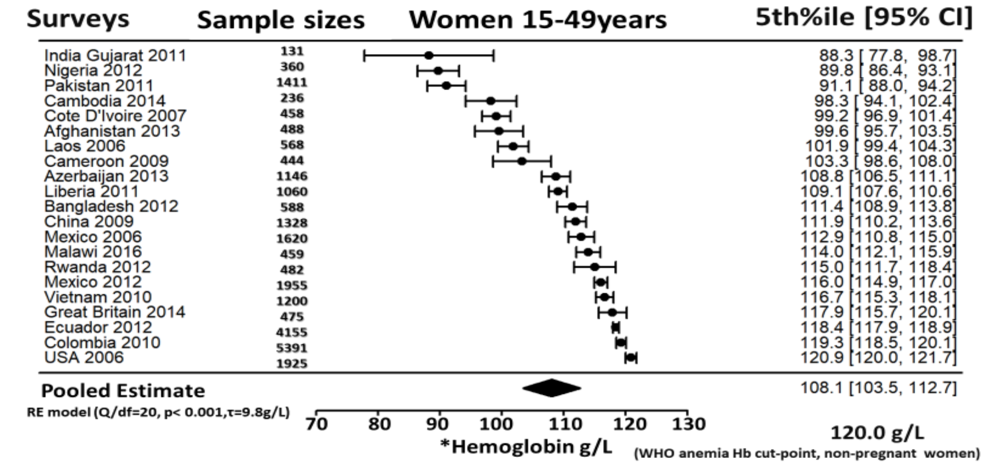
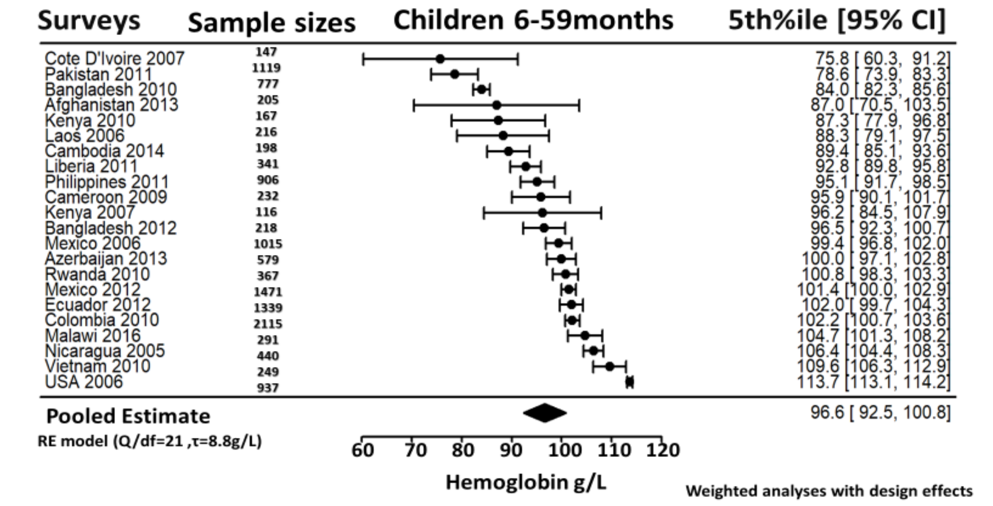	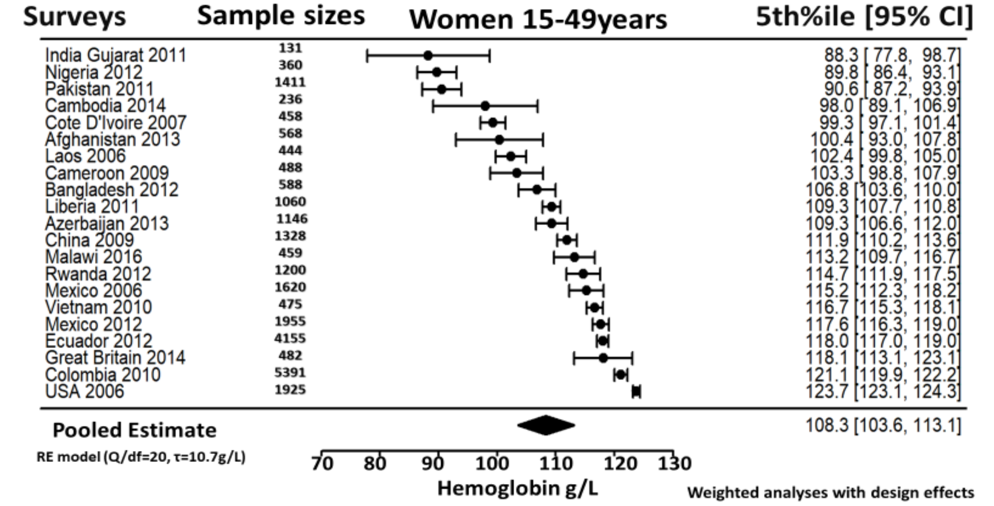
Test of equality of pooled effect sizes: 96.5 =96.6, g/L, p= 0.96	Test of equality of pooled effect sizes: 108.1 = 108.3 g/L, p= 0.94

Healthy defined as no inflammation (C-reactive protein ≤ 5 mg/dL [to convert to milligrams per liter, multiply by 10] or α-1-acid glycoprotein ≤ 1 g/L), no iron deficiency (based on ferritin <12 ng/mL for children and <15 ng/mL for women), no vitamin A deficiency (based on RBP or retinol ≥20.1 μg/dL [to convert to micromoles per liter, multiply by 0.0349], when available), and no known malaria. RE, random effects model. Q/df, Test of Cochrane's Q statistic for heterogeneity at the given degrees of freedom (DF). Standard errors, SE (and 95% CI) around Hb 5%ile were based on the Wald SE of the estimated proportion below the quantile at a design effect of 1 (for SRS). Hb values (to convert to grams per deciliter, divide by 10) were adjusted for attitude when available (Afghanistan, Azerbaijan, Colombia, Ecuador, Great Britain, Laos, Malawi, Mexico 2006 and 2012, and Rwanda) otherwise no adjustment was applied or <1000m so no adjustment needed. Hb values further adjusted for smoking among women (Colombia, Ecuador, Mexico 06 and 2012, Great Britain and USA).

© 2021 Addo OY et al. *JAMA Network Open.*

eTable 1: Blood Sampling, Biomarkers and Laboratory Assessment Methods Across Surveys

Survey	Population	Blood Sample	Biomarker and Laboratory Assessment Method						
			Hemoglobin	Retinol	Ferritin	sTfR	RBP	CRP	AGP
Afghanistan 2013	PSC, WRA	Venous	HemoCue® 201+	HPLC	TIA	N/A		Immunoassay	TIA
Azerbaijan 2013	PSC, WRA	Capillary	HemoCue® 201+	N/A	ELISA				
Bangladesh 2010	PSC	Capillary	HemoCue® 201+	N/A	ELISA				
Bangladesh 2012	PSC, WRA	Venous	HemoCue® 301	HPLC	ELISA	N/A		ELISA	
Burkina Faso 2010	PSC, WRA	Venous	HemoCue® 201+	HPLC	Turbidimetry	ELISA		Turbidimetry	Turbidimetry
Cambodia 2014	PSC, WRA	Capillary	HemoCue® 301	N/A	ELISA				
Cameroon 2009	PSC, WRA	Venous	HemoCue® 201+	HPLC	ELISA				
China 2009	WRA	Venous	Coulter™ AHA	N/A	RIA	Nephelometry	N/A	ITA	N/A
Colombia 2010	PSC, WRA	Capillary	HemoCue®	HPLC†	CLIA	CLIA	NA	Immunoassay	N/A
Cote d'Ivoire 2007	PSC, WRA	Venous	HemoCue® 201+	N/A	ELISA				
Ecuador 2012	PSC, WRA	Venous	AHA	HPLC	CLIA	N/A		Nephelometry	N/A
Great Britain 2014	WRA	Venous	Coulter™ AHA	HPLC	Immunoassay	EIA	N/A	PETIA	N/A
India Gujarat 2011	WRA	Venous	HemoCue®	N/A	ITA	ELISA	N/A	ITA	ITA
Kenya 2007	PSC	Capillary	HemoCue® Hb-B	N/A	ELISA				
Kenya 2010	PSC	Capillary	HemoCue® Hb-B	N/A	ELISA				
Laos 2006	PSC, WRA	Capillary	HemoCue® 201+	N/A	ELISA		N/A	Sandwich ELISA	
Liberia 2011	PSC, WRA	Capillary	HemoCue® Hb-B	N/A	ELISA				
Malawi 2016	PSC, WRA	Venous	HemoCue® 301	HPLC	ELISA				
Mexico 2006	PSC, WRA	Capillary	HemoCue®	N/A	Immunoassay	Immunoassay	N/A	Nephelometry	N/A
Mexico 2012	PSC, WRA	Capillary	HemoCue®	HPLC†	CMIA	N/A	N/A	Nephelometry	N/A
Mongolia 2006	PSC	Venous	HemoCue®	N/A	Immunoassay	N/A			Turbidimetry
Nigeria 2012	PSC, WRA	Venous	HemoCue® 201+	RP-HPLC	Sandwich ELISA				
Nicaragua 2005	PSC	Capillary	HemoCue® Hb-B	HPLC	Immunoassay	N/A			Turbidimetry
Papua New Guinea 2005	PSC, WRA	Capillary	HemoCue® 201+	N/A	N/A	ELISA			
Philippines 2011	PSC	Capillary	HemoCue® 201+	N/A	ELISA				
Pakistan 2011	PSC, WRA	Venous	HemoCue® 201+	HPLC	Turbidimetry	Unknown‡	N/A	Immunoassay‡	Turbidimetry
Rwanda 2010	PSC, WRA	Venous	HemoCue® 201+	N/A	ELISA				
United States 2003-06	PSC, WRA	Venous	Coulter™ AHA	RP-HPLC‡	IRMA	ITA	N/A	Nephelometry	N/A
Vietnam 2010	PSC, WRA	Venous	HemoCue® 301	RP-HPLC	ELISA	N/A	N/A	ELISA	N/A
Zambia 2009	PSC	Venous	HemoCue® 201+	HPLC	ELISA	N/A		RID	RID

Abbreviations: PSC, Preschool children 6-59 months. WRA, non-pregnant women of reproductive age 15-49 years, sTfR, soluble transferrin receptor. RBP, retinol binding protein. CRP, C-reactive protein. AGP, alpha-1acid glycoprotein. AHA, automated hematology analyzer. N/A, indicator not assessed in survey. CLIA, chemiluminescent immunoassay. CMIA, chemiluminescent microparticle immunoassay. EIA, enzyme immunoassay. ELISA, enzyme-linked immunosorbent assay. HPLC, high performance liquid chromatography. IRMA, immunoradiometric assay. ITA, immunoturbidimetric assay. PETIA, particle enhanced turbidimetric immunoassay. RIA, radioimmunoassay. RID, radial immunodiffusion. RP-HPLC, reversed phase chromatography. TIA, turbidimetric immunoassay. † Measured in PSC only. ‡ Measured in WRA only.

© 2021 Addo OY et al. *JAMA Network Open.*

eTable 2. Included Surveys and Participant Descriptive Characteristics: Preschool Children Aged 6-59 Months

**Survey/Country**	**Overall Study Sample (33,699) – No exclusions**								**Apparently Healthy** Analytic Sub-sample (13,445)**				
	**N**	**Age, Mo Mean (min-max)**	**Male %**	**ID %**	**VAD %**	**INFL %**	**Malaria %**	**Anemia %**	**N**	**Age, Mo Mean (min-max)**	**Male %**	**Anemia %**	**% Excluded**
**Afghanistan 2013**	601	28.5 (6.0,58.0)	53.2	20.0	52.6	26.5	NA	40.4	205	29.4 (6.4,58.0)	53.2	31.7	65.9
**Azerbaijan 2013**	1052	35.5 (6.0,59.0)	55.9	16.4	9.1	28.2	NA	24.9	579	37.1 (6.0,59.0)	53.5	16.1	45.0
**Bangladesh 2010**	1491	8.3 (6.0,11.0)	49.3	8.0	17.0	35.7	NA	83.5	777	8.3 (6.0,11.0)	47.7	79.2	47.9
**Bangladesh 2012**	435	36.9 (6.0,59.0)	54.0	13.3	28.7	29.2	NA	24.6	218	39.4 (6.0,59.0)	50.9	15.1	49.9
**Burkina Faso 2010****	124	48.5 (35.7,59.6)	48.4	0.0	39.5	91.9	15.0	71.8	2**	47.6 (35.9,59.3)	0.0	100.0	98.4
**Cote d'Ivoire 2007**	742	31.6 (6.0,59.0)	54.2	11.6	25.3	69.1	28.2	73.3	147	33.0 (6.0,59.0)	51.7	55.1	80.2
**Cambodia 2014**	408	36.1 (6.1,59.9)	55.6	4.4	10.3	39.7	--	52.2	198	37.3 (6.5,59.4)	52.0	47.0	51.5
**Cameroon 2009**	762	30.9 (12.0,59.9)	50.9	15.7	30.6	46.9	24.6	54.1	232	33.1 (12.0,59.9)	50.2	20.7	69.6
**Colombia 2010**	3792	37.9 (12.0,59.0)	52.8	10.2	25.8	19.1	NA	13.6	2115	39.6 (12.0,59.0)	53.2	11.1	44.2
**Ecuador 2012**	2015	30.0 (6.0,59.0)	50.5	9.4	20.9	12.2	NA	22.5	1339	31.7 (6.0,59.0)	49.4	15.2	33.5
**Kenya 2007**	873	20.0 (6.0,36.0)	52.8	38.8	23.0	65.5	19.9	66.9	116	20.4 (6.0,35.9)	48.3	33.6	86.7
**Kenya 2010**	843	21.5 (6.0,35.0)	50.2	19.2	29.5	61.8	32.5	71.5	167	22.1 (6.0,35.0)	47.3	43.7	80.2
**Laos 2006**	479	33.1 (6.0,59.0)	50.1	18.0	N/A	42.8	NA	40.3	216	37.6 (7.0,59.0)	44.9	35.2	54.9
**Liberia 2011**	1433	19.9 (6.0,35.9)	50.2	21.8	24.5	55.8	24.3	58.1	341	18.5 (6.0,35.9)	49.6	38.4	76.2
**Mongolia 2006****	194	20.1 (6.6,35.8)	52.6	36.1	36.6	25.8	NA	23.7	60**	21.9 (7.0,35.8)	50.0	13.3	69.1
**Malawi 2016**	1087	32.9 (6.0,59.0)	51.0	10.2	24.1	58.9	26.6	31.3	291	37.7 (6.0,59.0)	50.2	13.1	73.2
**Mexico 2006**	1590	40.9 (12.7,59.9)	52.3	26.2	N/A	11.2	NA	20.4	1015	42.4 (12.7,59.9)	51.4	16.9	36.2
**Mexico 2012**	2204	38.9 (12.0,59.9)	49.9	13.9	16.9	10.6	NA	16.9	1471	39.9 (12.0,59.9)	48.3	15.0	33.3
**Nigeria 2012****	546	30.7 (6.0,59.0)	50.1	5.1	42.9	63.2	35.2	74.7	85**	31.1 (10.0,59.0)	44.0	51.8	84.4
**Nicaragua 2005**	953	33.3 (6.1,59.9)	50.4	33.1	4.2	25.7	NA	16.6	440	36.6 (6.4,59.8)	49.3	11.1	53.8
**P. New Guinea 2005**¥	934	31.0 (6.0,59.8)	54.0	N/A	24.8	57.7	NA	50.1	NA	N/A	.	.	100.0
**Philippines 2011**	1767	14.8 (6.0,24.0)	50.1	23.0	8.3	27.9	NA	43.0	906	15.0 (6.0,24.0)	48.0	28.8	48.7
**Pakistan 2011**	6915	27.4 (6.0,59.0)	52.0	46.1	53.2	35.0	NA	60.5	1119	29.3 (6.0,59.0)	50.3	46.2	83.8
**Rwanda 2010**	576	34.7 (6.0,59.0)	47.5	5.2	11.1	28.8	NA	27.1	367	35.6 (6.0,59.0)	45.3	19.6	36.3
**United States 2003-06**	1135	35.8 (12.0,59.0)	50.6	11.2	N/A	6.4	NA	3.1	937	37.1 (12.0,59.0)	50.2	1.6	17.4
**Vietnam 2010**	360	37.3 (10.4,59.7)	52.9	13.9	6.9	12.8	NA	7.5	249	39.2 (12.7,59.7)	52.2	4.0	30.8
**Zambia 2009****	388	35.3 (6.0,59.6)	58.2	4.1	56.7	76.0	18.8	51.8	50**	33.5 (6.4,59.5)	54.0	22.0	87.1

Abbreviations and definitions: Anemia based on hemoglobin (Hb) <110 g/L after Hb adjusted for attitude when available (Afghanistan, Azerbaijan, Colombia, Ecuador, Laos, Malawi, Mexico 2006 and 2012, and Rwanda) otherwise no adjustment was applied or <1000 m was assumed. ID, iron deficiency based on ferritin <12 ng/mL; N/A, indicator not applicable; VAD, vitamin A deficiency based on retinol binding protein (RBP) or retinol <20.1 μg/dL (to convert to micromoles per liter, multiply by 0.0349), INFL, inflammation based on C-reactive protein > 0.5 mg/dL (to convert to milligrams per liter, multiply by 10) or α-1-acid glycoprotein >1 g/L. ** Healthy defined as no inflammation, ID, VAD or malaria, if measured. Surveys dropped for Hb threshold analyses due to <100 "healthy" individuals. The Hb-sTfR curve analyses was based on data from all 17 surveys for children and 17 surveys for women. The Hb threshold analyses were based on 22 surveys or 21 countries except for Burkina Faso, Mongolia, Nigeria, Papua N Guinea, and Zambia, which did not meet the inclusion criteria or did not have enough (>100) sample sizes after the exclusions.

© 2021 Addo OY et al. *JAMA Network Open.*

eTable 3. Included Surveys and Participant Descriptive Characteristics: Non-Pregnant Women of Reproductive Aged 15-49 y

Survey/Country	Overall Study Sample (46,251) – No exclusions							Apparently Healthy** Analytic Sub-sample (25,880)			
	N	Age, Yr. Mean (min-max)	ID %	VAD %	INFL %	Malaria %	Anemia %	N	Age, Yr. Mean (min-max)	Anemia %	% Excluded
**Afghanistan 2013**	950	30.9 (15.0,49.0)	26.6	11.5	20.3	N/A	41.9	488	30.8 (15.0,49.0)	34.6	48.6
**Azerbaijan 2013**	2650	32.2 (15.0,49.9)	31.7	0.6	33.5	N/A	36.6	1146	31.3 (15.0,49.9)	18.8	56.8
**Bangladesh 2012**	865	29.2 (15.0,49.0)	8.8	8.7	19.2	N/A	23.4	588	29.4 (15.0,49.0)	17.5	32.0
**Burkina Faso 2010****	127	31.7 (20.0,49.0)	3.1	14.2	76.4	61.1	39.4	17**	31.4 (20.0,43.0)	41.2	86.6
**Cote d'Ivoire 2007**	828	27.9 (15.0,48.0)	12.9	0.8	32.9	4.8	50.6	458	28.2 (15.0,48.0)	42.4	44.7
**Cambodia 2014**	433	30.1 (16.0,49.0)	2.3	3.7	41.1	N/A	43.2	236	30.3 (16.0,48.0)	42.8	45.5
**Cameroon 2009**	749	27.1 (15.0,47.5)	14.4	2.1	20.3	14.4	37.4	444	27.2 (15.0,47.5)	25.0	40.7
**China 2009**	1954	37.7 (15.0,49.0)	27.7	N/A	5.3	N/A	19.9	1328	37.8 (15.0,49.0)	11.7	32.0
**Colombia 2010**	9066	29.0 (15.0,49.9)	22.2	N/A	22.2	N/A	8.1	5391	28.4 (15.0,49.9)	4.7	40.5
**Ecuador 2012**	5976	33.3 (19.0,49.0)	14.0	2.1	17.1	N/A	13.3	4155	33.1 (19.0,49.0)	5.9	30.5
**Great Britain 2014**	706	32.7 (15.0,49.0)	19.3	0.6	16.0	N/A	10.6	475	33.0 (15.0,49.0)	5.7	32.7
**Gujarat India 2011**	323	25.4 (15.0,45.0)	45.8	N/A	19.5	N/A	94.7	131	25.6 (15.0,45.0)	92.4	59.4
**Liberia 2011**	863	29.3 (15.0,49.0)	22.9	N/A	13.7	N/A	37.3	568	29.5 (15.0,49.0)	23.4	34.2
**Laos 2006**	1942	28.2 (15.0,49.9)	19.4	2.2	18.7	15.4	33.2	1060	29.2 (15.0,49.5)	28.6	45.4
**Malawi 2016**	766	28.4 (15.0,49.0)	14.2	2.1	16.1	15.1	21.5	459	29.1 (15.0,49.0)	12.0	40.1
**Mexico 2006**	3020	31.1 (15.1,50.0)	26.6	N/A	24.9	N/A	15.0	1620	29.8 (15.1,50.0)	9.2	46.4
**Mexico 2012**	3612	35.1 (15.6,50.0)	27.5	N/A	21.8	N/A	14.6	1955	34.8 (15.6,50.0)	8.5	45.9
**Nigeria 2012**	618	27.3 (15.0,49.0)	8.6	5.0	26.2	9.8	54.7	360	27.5 (15.0,45.0)	52.5	41.7
**Papua New Guinea 2005[¥]**	779	29.3 (15.0,49.0)	N/A	0.6	23.7	N/A	40.0	NA	N/A	.	100.0
**Pakistan 2011**	4839	30.8 (16.0,49.0)	34.7	36.9	30.0	N/A	49.1	1411	31.0 (16.0,49.0)	36.5	70.8
**Rwanda 2010**	596	31.1 (18.0,45.0)	3.9	2.2	15.3	N/A	10.2	482	31.1 (18.0,45.0)	7.3	19.1
**United States 2003-06**	3097	28.9 (15.0,49.9)	16.0	0.3	24.2	N/A	9.4	1925	27.8 (15.0,49.9)	3.3	37.8
**Vietnam 2010**	1492	32.3 (15.0,49.0)	13.1	N/A	6.6	N/A	11.4	1200	32.5 (15.0,49.0)	7.0	19.6

Abbreviations and definitions: Anemia based hemoglobin (Hb) <120 g/L after Hb adjusted for 1) attitude when available (Afghanistan, Azerbaijan, Colombia, Ecuador, Great Britain, Laos, Malawi, Mexico 2006 and 2012, and Rwanda) otherwise no adjustment was applied or <1000m is assumed and no adjustment needed, and 2) smoking (Colombia, Ecuador, Mexico 06 and 2012, Great Britain and USA). ID, iron deficiency based on ferritin <15 ng/mL; N/A, indicator not assessed in survey; VAD, vitamin A deficiency based on retinol binding protein (RBP) or retinol <20.1 µg/dL (to convert to micromoles per liter, multiply by 0.0349), when available., INFL, inflammation based on C-reactive protein (CRP) > 0.5 mg/dL (to convert to milligrams per liter, multiply by 10) or α-1-acid glycoprotein (AGP) >1 g/L. **Healthy defined as no inflammation, ID, VAD or malaria, if measured. Surveys dropped for Hb threshold analyses due to <100 "healthy" individuals. The Hb-sTfR curve analyses was based on data from all 17 surveys for each target group. The Hb threshold analyses were based on 21 surveys or 20 countries except for Burkina Faso and Papua New Guinea (lacked ferritin) which did not meet the inclusion criteria or did not have enough (>100) sample sizes after the exclusions.

© 2021 Addo OY et al. *JAMA Network Open.*

eTable 4: Results of One-Way Quantile Test Comparing Survey-Specific 5th Percentile Hb With Current WHO Hb Cutoffs for Defining Anemia in a Multinational Sample of Apparently Healthy Individuals

Preschool Children)	5TH%ile Hb 95%CI	Ho: Survey 5th%ile Hb = 110 g/L (WHO anemia cutoff*)	Non-Pregnant Women	5TH%ile Hb 95%CI	Ho: 5th%ile Hb = 120 g/L (WHO anemia cutoff*)
		P			P
Pakistan 2011	79.0 (75.4,82.6)	<.001	Indian Gujarat 2011	88.3 (77.8,98.7)	<.001
Cote D'Ivoire 2007	79.4 (66.2,92.6)	<.001	Nigeria 2012	89.8 (86.4,93.1)	<.001
Bangladesh 2010	84.0 (82.3,85.6)	<.001	Pakistan 2011	91.1(88.0,94.2)	<.001
Kenya 2010	87.3 (77.9,96.8)	<.001	Cambodia 2014	98.3 (94.1,102.4)	<.001
Afghanistan 2013	88.2 (82.0,94.5)	<.001	Cote D'Ivoire 2007	99.2 (96.9,101.4)	<.001
Cambodia 2014	88.9 (85.1,92.8)	<.001	Afghanistan 2013	99.6 (95.7,103.5)	<.001
Laos 2006	90.6 (85.5,95.7)	<.001	Laos 2006	101.9 (99.4,104.3)	<.001
Liberia 2011	91.5 (87.9,95.1)	<.001	Cameroon 2009	103.3 (98.6,108.0)	<.001
Philippines 2011	95.3 (92.8,97.7)	<.001	Azerbaijan 2013	108.8 (106.5,111.1)	<.001
Kenya 2007	96.2 (84.5,107.9)	<.001	Liberia 2011	109.1 (107.6,110.6)	<.001
Cameroon 2009	97.6 (93.0,102.2)	<.001	Bangladesh 2012	111.4 (108.9,113.8)	<.001
Mexico 2006	98.0 (96.0,99.9)	<.001	China 2009	111.9 (110.2,113.6)	<.001
Azerbaijan 2013	98.2 (95.5,101.0)	<.001	Mexico 2006	112.9 (110.8,115.0)	<.001
Rwanda 2010	100.7 (98.1,103.3)	<.001	Malawi 2016	114.0 (112.1,115.9)	<.001
Mexico 2012	100.8 (99.5,102.0)	<.001	Rwanda 2010	115.0 (111.7,118.4)	<.001
Bangladesh 2012	101.5 (98.1,104.9)	<.001	Mexico 2012	116.0 (114.9,117.0)	<.001
Ecuador 2012	102.0 (101.2,102.8)	<.001	Vietnam 2010	116.7 (115.3,118.1)	<.001
Colombia 2010	102.7 (101.7,103.7)	<.001	Great Britain 2014	117.9 (115.7,120.1)	.031
Malawi 2016	103.3 (99.7,106.8)	<.001	Ecuador 2012	118.4 (117.9,118.9)	<.001
Nicaragua 2005	105.2 (104.0,106.5)	<.001	Colombia 2010	119.3 (118.5,120.1)	.69
Vietnam 2010	109.6 (106.3,113.0)	.65	USA 2006	120.9 (120.0,121.7)	.17
USA 2006	112.3(111.4,113.3)	.99			

*Population used did not exclude individuals with nutritional deficiencies, inflammation and or malaria (if measured). %ile - percentile. To convert Hb to grams per deciliter, divide by 10.

© 2021 Addo OY et al. *JAMA Network Open.*

eTable 5: Associations Between Hb Concentrations With Participant Age, Hb Analyzer, Type of Blood Sampling in a Multinational Sample of Apparently Healthy Individuals.

	**Preschool Children 6-59mo(N=13,445)**		**Non-Pregnant Women 15-49y (N=25,880)**		
**Fixed Effects**	**β (SE), g/L**	**P**	**Fixed Effects**	**β (SE), g/L**	**P**
Age(months) categories			Age(years) categories		
6-11	-9.2 (-10.2, -8.3)	<.01	15-19	-0.8 (-1.4, -0.1)	0.02
12-18	-7.1 (-7.9, -6.3)	<.01	20-24	0.0* (-0.6,0.6)	0.99
18-23	-5.3 (-6.2, -4.5)	<.01	25-29	-0.6 (-1.2,0.0)	0.06
24-35	-4.0 (-4.6, -3.4)	<.01	30-34	-1.1 (-1.7, -0.5)	<0.01
36-47	-1.3 (-1.9, -0.7)	<.01	35-39	-0.1 (-0.7,0.5)	0.76
Ref (>=48mo)	-	-	40-44	-0.9 (-1.6, -0.3)	<0.01
Child sex (ref: female)	0.1 (-0.3,0.5)	0.65	Ref (>=45)	-	-
Hemocue® v. automated hematology analyzer	-8.2 (-19.9,3.5)	0.17	Hemocue® v. automated Hematology analyzer	-15.5 (-35.2,4.1)	0.12
Hemocue® 201+ (v. non Hemocue 201+)	-1.1 (-8.2,6.1)	0.77	Hemocue® 201+ (ref: non Hemocue 201+)	-6.1 (-23.4,11.1)	0.49
Hemocue® 301 (v. non Hemocue 301)	-3.0 (-12.4,6.4)	0.58	Hemocue® 301 (ref: non Hemocue 301)	-8.2 (-27.1,10.6)	0.39
Venous v. Capillary	-1.7 (-7.7,4.4)	0.59	Venous v. Capillary	7.8 (-0.5,16.1)	0.07
**Variance decomposition (for Random effects)**	%		**Variance decomposition (for Random effects)**	%	
ICC: between surveys	19.3	<0.01	ICC: between surveys	30.0	<0.01
ICC: between participants across all surveys	80.7		ICC: between participants across all surveys	70.0	
Model adjusted *conditional pseudo R^2	25.9 %		Model adjusted *conditional pseudo R^2	33.7%	-

* conditional pseudo R^2 based on both fixed and random effects. Age was mean centered across surveys. Healthy defined as no inflammation (CRP ≤ 0.5 mg/dL [to convert to milligrams per liter, multiply by 10] or α-1-acid glycoprotein ≤ 1 g/L), no iron deficiency (based on ferritin <12 ng/mL for children and <15 ng/mL for women), no vitamin A deficiency (based on RBP or retinol ≥20.1 µg/dL [to convert to micromoles per liter, multiply by 0.0349]), and no known malaria. Estimates calculated from generalized linear mixed models using survey as random intercept. Hb values were adjusted for attitude when available (Afghanistan, Azerbaijan, Colombia, Ecuador, Great Britain, Laos, Malawi, Mexico 2006 and 2012, and Rwanda) otherwise no adjustment was applied or <1000m is assumed. Hb values further adjusted for smoking among women from (Colombia, Ecuador, Mexico 2006 and 2012, Great Britain and USA).*Values non-zero but rounds to 0.00 at 2 decimal places.

© 2021 Addo OY et al. *JAMA Network Open.*
